# Genomic comparative analysis and gene function prediction in infectious diseases: application to the investigation of a meningitis outbreak

**DOI:** 10.1186/1471-2334-13-554

**Published:** 2013-11-19

**Authors:** Enrico Lavezzo, Stefano Toppo, Elisa Franchin, Barbara Di Camillo, Francesca Finotello, Marco Falda, Riccardo Manganelli, Giorgio Palù, Luisa Barzon

**Affiliations:** 1 Department of Molecular Medicine, University of Padova, Padova, Italy; 2 Regional Reference Laboratory for Infectious Diseases, Microbiology and Virology Unit, Padova University Hospital, Padova, Italy; 3 Department of Information Engineering, University of Padova, Padova, Italy

**Keywords:** *Neisseria meningitidis*, Whole genome sequencing, Next generation sequencing, capsule locus, Comparative genomics, 454 pyrosequencing, Meningitis outbreak, Molecular epidemiology, Gene function prediction

## Abstract

**Background:**

Next generation sequencing (NGS) is being increasingly used for the detection and characterization of pathogens during outbreaks. This technology allows rapid sequencing of pathogen full genomes, useful not only for accurate genotyping and molecular epidemiology, but also for identification of drug resistance and virulence traits.

**Methods:**

In this study, an approach based on whole genome sequencing by NGS, comparative genomics, and gene function prediction was set up and retrospectively applied for the investigation of two *N. meningitidis* serogroup C isolates collected from a cluster of meningococcal disease, characterized by a high fatality rate.

**Results:**

According to conventional molecular typing methods, all the isolates had the same typing results and were classified as outbreak isolates within the same *N. meningitidis* sequence type ST-11, while full genome sequencing demonstrated subtle genetic differences between the isolates. Looking for these specific regions by means of 9 PCR and cycle sequencing assays in other 7 isolates allowed distinguishing outbreak cases from unrelated cases. Comparative genomics and gene function prediction analyses between outbreak isolates and a set of reference *N. meningitidis* genomes led to the identification of differences in gene content that could be relevant for pathogenesis. Most genetic changes occurred in the capsule locus and were consistent with recombination and horizontal acquisition of a set of genes involved in capsule biosynthesis.

**Conclusions:**

This study showed the added value given by whole genome sequencing by NGS over conventional sequence-based typing methods in the investigation of an outbreak. Routine application of this technology in clinical microbiology will significantly improve methods for molecular epidemiology and surveillance of infectious disease and provide a bulk of data useful to improve our understanding of pathogens biology.

## Background

The recent success of next generation sequencing technologies (NGS), that are now available in many laboratories and sequencing centers, is deeply impacting our capability to investigate biological samples in many fields of research, including microbiology and virology [1,2]. In this regard, the main applications fostered by NGS are the identification of novel pathogens, the metagenomics analysis of biological and environmental niches, the investigation of transcriptomes, and the characterization of full-length microbial and viral genomes for a wide range of purposes, including phylogenetic and epidemiological studies and the characterization of drug-resistant or immune-escaping mutants [3-6].

For each of these applications, a plethora of bioinformatics tools is available, both academic and commercial, that has to be applied and coordinated depending on the specific needs. In this study we report the application of an analysis pipeline which combines genomic comparative analysis and gene function prediction to finely characterize microbial genomes. The pipeline was applied to the retrospective analysis of a group of *Neisseria meningitidis* isolates collected during an outbreak. Epidemiological, clinical, and surveillance data on this outbreak, which occurred in north-eastern Italy during the 2007–2008 winter and was characterize by a high fatality rate, have been previously reported [7,8]. The results of this study highlight the essential contribution of whole genome sequencing, performed by NGS technology, to distinguish outbreak cases, i.e., related cases with a common epidemiological source, from clusters of temporary and geographically proximate but unrelated cases. In addition, genomic comparative analysis and gene function prediction led to the identification of genetic changes in the capsule locus that could have contributed to pathogenicity.

## Methods

### *N. meningitidis* isolates

*N. meningitidis* isolates of an outbreak which occurred in Veneto Region (north-eastern Italy) in December 2007-January 2008 were collected by local hospital laboratories and sent to the Regional Reference Laboratory at Padua University Hospital for confirmation, phenotypic characterization, and molecular typing. The outbreak strains analyzed in the present study included *N. meningitidis* isolates from seven patients (mean age 23 year, range 15–33 years) from a relatively small geographical area, who had disease onset between December 13, 2007 and January 4, 2008.

The study was approved by the Ethics Committee of Padova University Hospital (protocol no. 53503).

### Phenotypic and genotypic characterization of *N. meningitidis* isolates

Serogrouping, which was performed by slide agglutination using commercial antisera (Remel Europe Ltd, Dartford, UK), classified all the 7 isolates as serogroup C. All isolates were fully susceptible to penicillin, rifampicin, ceftriaxone, and ciprofloxacin. Pulsed-field gel electrophoresis analysis gave the same electrophoresis pattern for all isolates, indicating their relatedness. Molecular characterization by MLST, performed according to Maiden *et al*. [9], demonstrated that all isolates belonged to the same sequence type and clonal complex ST-11 (all isolates had the following MLST alleles: *abcZ* 2, *adk* 3, *aroE* 4, *fumC* 3, *gdh* 8, *pdhC* 4, *pgm* 6). In addition, sequencing of *N. meningitidis PorA* variable regions 1 and 2 [10] confirmed that all isolates had the same *PorA* subtype 5–1, 10–8.

### Whole genome sequencing of *N. meningitidis* isolates by 454 pyrosequencing

Whole genome sequencing of two *N. meningitidis* isolates, the index case (named K1207) and the last case (named S0108) of the outbreak, was performed with the objective of confirming their relatedness and to detect genetic differences between the two strains that could have occurred during the short period of the outbreak. The draft genome sequences of these two *N. meningitidis* isolates were reported in a previous announcement [11].

Genomic DNA was purified from meningococcal isolates using a phenol-chloroform-based method.

Sequencing was performed using a Roche 454 Life Sciences Genome Sequencer FLX platform following the manufacturer’s instructions (Roche 454 Life Sciences, Branford, CT, USA). For each sample, 2 different libraries were prepared, a shotgun and a 3 kb paired-end, starting from 5 μg of genomic DNA. The shotgun library was prepared as follows: after nebulization, purification and adaptors ligation, DNA fragments were clonally amplified using the Emulsion PCR Kit I (Roche). For the preparation of the paired end library, genomic DNA was fragmented by hydrodynamic shearing, followed by a size selection step, hairpin adaptors ligation and circularization of fragments. From this step the procedure was similar to the shotgun one, consisting in the nebulization of circular molecules, paired end adaptors ligation and amplification of the library. The clonal amplification was carried out using the Emulsion PCR Kit II (Roche). Sequencing was performed on a GS FLX instrument, using the Standard LR70 Sequencing Kit (Roche). Images were processed using the runAnalysisPipe and runAnalysisPipePairedEnd commands provided with the DataProcessing package (Roche). With respect to the previous genome announcement [11], we performed a new *de novo* assembly with the most recent version of the Newbler software (v.2.6), which is more effective and produces a smaller number of contigs with respect to older versions [12].

### Gene identification and comparison between the two *N. meningitidis* genomes and comparison with FAM18 reference genome

The assembled sequences of K1207 and S0108 isolates were compared to each other and with the most similar genome among those already available in RefSeq database, i.e., the FAM18 strain genome (NC_008767). Whole genome level comparison was made with MUMmer3.23 package, to investigate possible large recombination events.

To compare the CDS content between K1207 and S0108 genomes an approach to find the reciprocal blast best hits was implemented. This method is widely used to identify the coding sequences that are supposed to be “orthologs”. The following steps were performed:

download of protein sequences of genes annotated on each of the following complete *N. meningitidis* genomes, spanning different serogroups: FAM18 (NC_008767) and 053442 (NC_010120) from serogroup C, Z2491 (NC_003116) from serogroup A, MC58 (NC_003112) from serogroup B and the capsule null strain alpha14 (NC_013016), for which the serogroup cannot be defined.

clustering of protein sequences from the previous step at 90% of sequence identity, using cd-hit [13,14], in order to eliminate redundancy and create a reference proteome.

*de novo* gene prediction on isolates K1207 and S0108 using Glimmer3.02 [15] and extraction of the corresponding protein sequences.

search of protein sequences belonging to each isolate against this reference proteome using blastp [16]; since all analyzed protein sequences belong to the same species, only matches with at least 90% of sequence identity and covering at least 90% of sequence length were considered. The threshold was chosen after testing different cutoff values: relaxing this parameter did not produce substantial changes in the number of shared CDSs, while tightening the percentage identity cutoff resulted in a rapid decrease in the number of shared CDSs (see Additional file 1: Figure S1). Nonetheless, this high stringency led to the loss of many similar genes whose function is identical.

starting from blastp output, a table of matches was created in which, for each protein, “0” or “1” indicated its absence or presence in the different genomes. From this table it was possible to extract different lists of genes, common to both genomes, or specific to one or another.

To better characterize the differences between the two isolates, function prediction of unknown genes was done:

genes with unknown function were analyzed by means of the function prediction tool Argot2 [17-19]. This algorithm was developed by our bioinformatics group and allows to retrieve scored GO annotations for a given gene or CDS, starting from its nucleotide or protein sequence.

**Figure 1:**
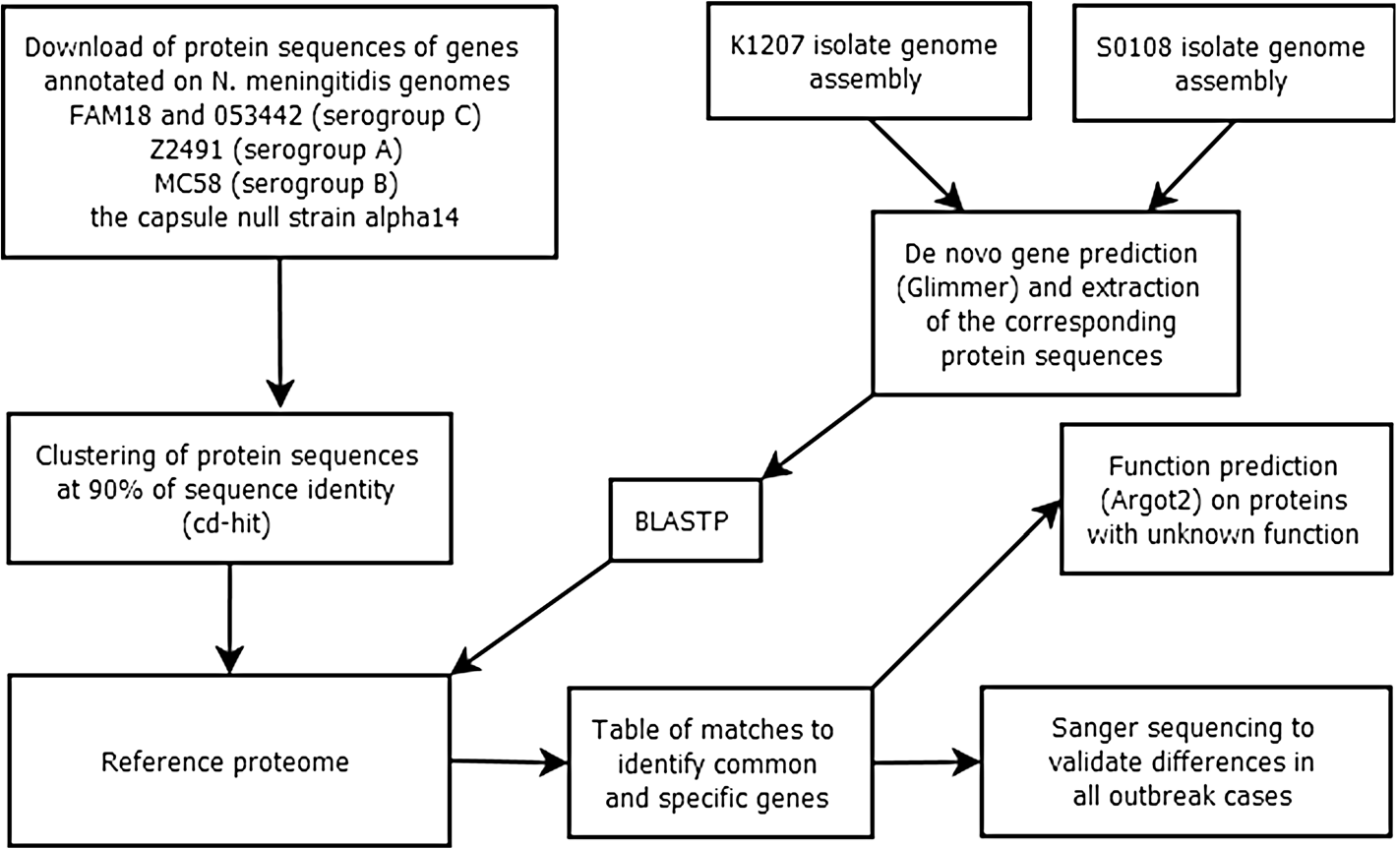
**Analysis algorithm.** Diagram of the analysis workflow applied to the investigation of a meningitis outbreak based on genomic comparative analysis and gene function prediction.

The workflow of the analysis pipeline is summarized in Figure 1.

### Validation of genetic differences among *N. meningitidis* isolates by target-specific PCR and cycle sequencing

Some of the genes found to be present in only one of the two *N. meningitis* isolates were selected for validation with target-specific PCR and conventional cycle sequencing. A detailed list of investigated genes is reported in Table 1, along with brief gene function descriptions. These genes were also screened in the other five *N. meningitidis* isolates from the outbreak and in sporadic *N. menigitidis* isolates, with identical multilocus sequence type and *porA* genotype, which were collected in Veneto Region in 2007 and 2008.

**Table 1:** Gene-specific PCR and Sanger sequencing validation of differences detected by NGS

	***N. meningitidis *** **serogroup C isolates**								
**CDS ID**	**A**	**K1207**	**B**	**C**	**D**	**E**	**F**	**S0108**	**G**
	**Nov 2007**	**Dec 2007**	**Dec 2007**	**Dec 2007**	**Dec 2007**	**Dec 2007**	**Dec 2007**	**Jan 2008**	**Apr 2008**
**K1207-specific CDSs**									
GI:352289428 (SacB, capsular polysaccharide phosphotransferase)	-	+	+	+	+	+	+	-	-
GI:254805876 (putative endonuclease)	-	+	+	+	+	+	+	-	-
GI:388935 (TDP-deoxymannose-dehydratase)	-	+	+	+	+	+	+	-	-
GI:254673887 (lipA5, capsule polysaccharide modification protein)	-	+	+	+	+	+	+	-	-
GI:254671124 (hypothetical protein)	-	+	+	+	+	+	+	-	-
**S0108-specific CDSs**									
GI:121635673 (putative pilin)	+	-	-	-	-	-	-	+	+
GI:254673874 (hypothetical protein)	+	-	-	-	-	-	-	+	+
GI:254671515 (LipA, Capsule polysaccharide export protein)	+	-	-	-	-	-	-	+	+
GI:385323246 (hypothetical protein)	+	-	-	-	-	-	-	+	+
**MLST**	ST-11/ET-37	ST-11/ET-37	ST-11/ET-37	ST-11/ET-37	ST-11/ET-37	ST-11/ET-37	ST-11/ET-37	ST-11/ET-37	ST-11/ET-37
**PorA VR1/2**	P1.5-1, 10-8	P1.5-1,10-8	P1.5-1,10-8	P1.5-1,10-8	P1.5-1,10-8	P1.5-1,10-8	P1.5-1,10-8	P1.5-1,10-8	P1.5-1,10-8

+ present, by PCR; - not present, by PCR.

The target CDSs that were specifically detected in the K1208 and S0108 genomes by 454 pyrosequencing were validated by means of PCR and Sanger sequencing. Then, they were searched also in *N. meningitidis* isolates A-G, which represent all the *N. meningitidis* serogroup C isolates classified as ST-11/ET-15 that were collected in Veneto Region in 2007–2008. Isolates B-F show the same pattern of K1207 and are classified as “outbreak cases”; isolates A and G are sporadic cases and are similar to S0108.

## Results

### Pyrosequencing results

The index and the last cases of a *Neisseria meningitidis* outbreak, named K1207 and S0108 respectively, were sequenced in a 454 FLX platform. For K1207 genome, a total of 257,909 shotgun reads were obtained, with average length of 237 bases, and 63,593 paired end reads with an average distance of 3,113 nucleotides. For genome S0108, the shotgun reads obtained were 241,983 with an average length of 235 bases, while paired end reads were 50,658 with an average distance of 2560 bases. The average depth was 27 to 28 fold for both genomes. Raw reads were assembled with Newbler2.6.

### Characteristics of sequenced genomes and plasmids of *N. meningitidis* isolates

The genome sequences of the two *N. meningitidis* serogroup C ST-11 isolates were very similar to *N. meningitidis* serogroup C FAM18 strain, which is a representative of the ST-11/ET-37 clonal complex and whose sequence is completely finished and deposited in RefSeq (NC_008767). A deeper analysis revealed that the two isolates belonged to the ET-15 variant of the ST-11 complex, as demonstrated by the presence of the IS*1301* insertion element [20] and the ET-15 fumarate hydratase gene [21].

The sequence of a 7-kb plasmid, identical in both isolates and very similar to pJS-B (RefSeq accession number NC_004758) was also obtained. Draft genome sequences have been deposited in GenBank (accession numbers ADWM02000000 and ADWN02000000 for K1207 and S0108, respectively).

### Comparison of *N. meningitidis* serogroup C genomes through analysis of their coding sequence content

To screen for the presence of genetic variations between the two isolates, the K1207 and S0108 genomes were compared by assessing their coding sequence (CDS) content, looking for shared and genome-specific CDSs. The latter were defined as CDSs for which a reciprocal match between the two isolates was not detected. Following the criteria described in the methods section, 2011 CDSs were identified as present in both genomes, 11 CDSs were exclusively present in K1207 genome, while 10 CDSs were present in S0108 genome only. Since most unique CDSs encoded hypothetical proteins of unknown function, we performed a function prediction step by using Argot^2^ webservice, an in-house developed tool [17-19]; the complete report of predicted functional annotations is available in Additional file 1: Figure S1. These genes were predicted to encode proteins belonging to the membrane compartment, involved in capsular polysaccharide biosynthetic process, or adhesins and transport proteins.

### Screening for genetic differences in other *N. meningitidis* serogroup C ST-11 isolates of the outbreak

Since the dissimilarities between the two *N. meningitidis* isolates could be the result of genetic variations of the same strain within the outbreak, we selected a subset of these differences for further investigation and validation by using target-specific PCR and cycle sequencing. This analysis was performed in the two sequenced *N. meningitidis* K1207 and S0108 strains for confirmation and in the other five isolates of the outbreak (i.e., isolates B-F in Table 1), as well as in other two ST-11 isolates (i.e., isolates A and G in Table 1), which were collected from sporadic meningitis cases that occurred in the same area in November 2007 and in April 2008. The results of this analysis confirmed findings provided by NGS and bioinformatics comparison for the K1207 and the S0108 isolates and, unexpectedly, they demonstrated that the S0108 isolate did not belong to the outbreak cluster. In fact, while the other five isolates from the same outbreak shared the same sequences of the outbreak index case K1207, the CDSs of S0108 were found in the strains isolated from sporadic meningitis cases, but differed from those of the outbreak isolates (Table 1). Thus, these results demonstrate that the outbreak was caused by the K1207 strain, while the S0108 strain was circulating in the same region during the same season and caused sporadic meningitis cases.

### Analysis of the capsule locus

**Figure 2:**
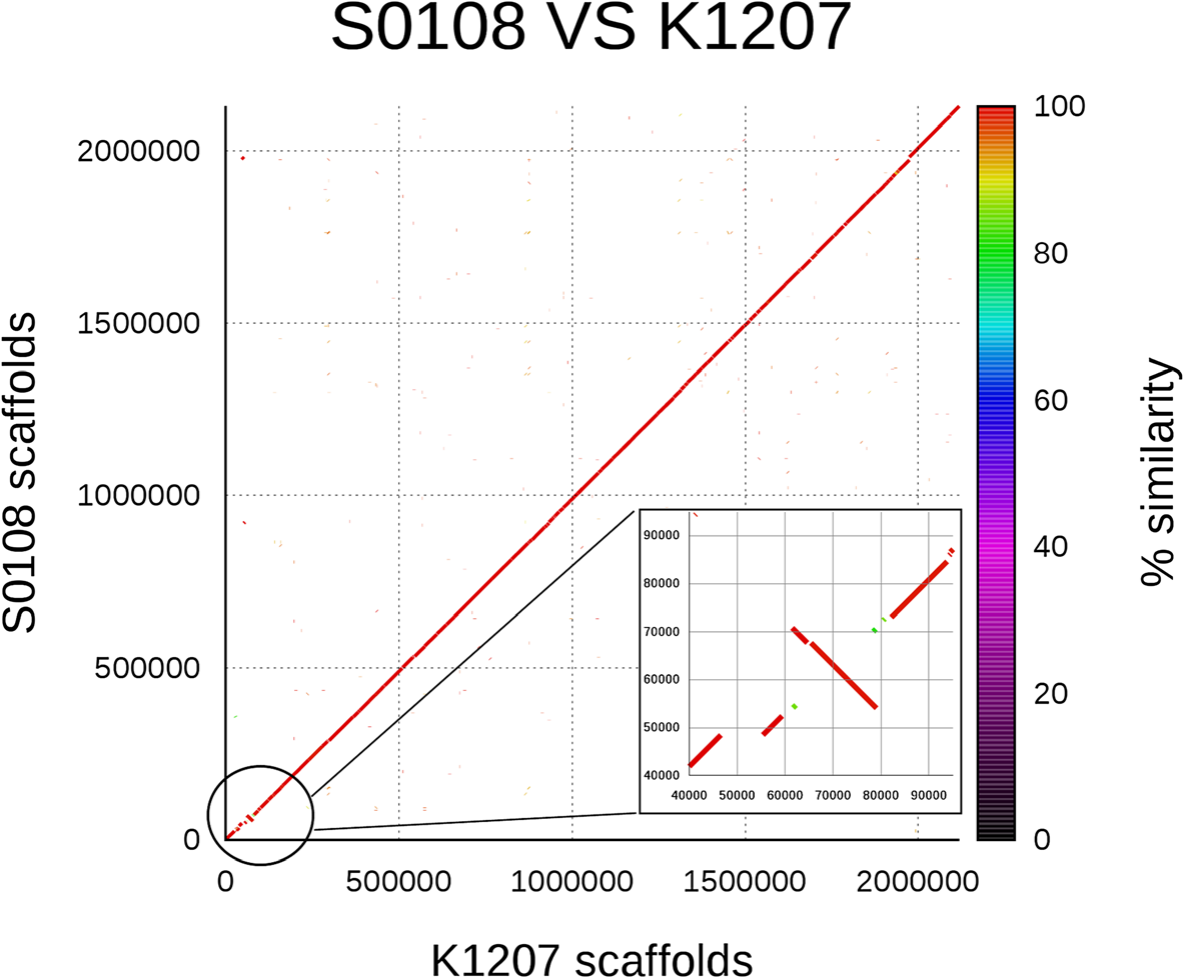
**Genome-wide comparison of samples K1207 and S0108.** Whole genome alignment between isolate K1207 vs. isolate S0108. The inset highlights the 20 Kb inversion in the capsule locus region between the two isolates. The dot plot was generated with MUMmer3.23 (http://mummer.sourceforge.net) and indicates matching sequences in forward and reverse direction.

**Figure 3:**
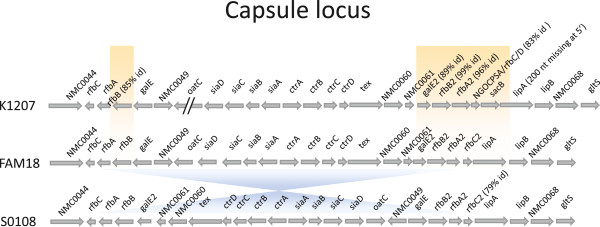
**Capsule locus modifications.** Map of the capsule locus of K1207 and S0108 *N. meningitidis* isolates in comparison with FAM18 strain. Changes in CDS content between K1207 and FAM18 are highlighted in yellow, with the percentage similarity of amino acid sequences reported in parenthesis; the 20 kb inversion in the S0108 genome is indicated in blue.

Comparison among K1207, S0108, and FAM18 *N. meningitidis* genomes at whole genome level and for CDS content demonstrated that most genetic variations occurred in the capsule locus. The results of whole genome comparison between K1207 and S0108 are illustrated in Figure 2, where an inversion in the capsule locus of S0108 is demonstrated. The whole genome alignment is shown with a dot plot matrix, where a dot is drawn each time there is a match between two regions of the genomes. Ideally, a perfect alignment would result in a continuous diagonal line from the start to the end of genomes; the presence of a chromosome inversion is highlighted by a break in the diagonal, with the change in dots direction indicating an inverse complement alignment. A map summarizing genetic changes among K1207, S0108 and FAM18 genomes is shown in Figure 3, where the blue chiasma indicates the same inversion present in S0108 with respect to FAM18.

The K1207 genome had remarkable changes in CDS content and nucleotide sequence of the capsule locus in comparison with both S0108 and FAM18 strains, which were consistent with multiple events of recombination and horizontal gene transfer. Genetic variations in the capsule locus of K1207 affected (i) the *refB* gene, which had a deletion in 3′, (ii) the duplicated *galE-rfbBAC* operon (for lipooligosaccharide biosynthesis) and the downstream *lipAB* operon (for phospholipid modification of the capsular polysaccharide, required for its translocation to the cell surface), which had genes with markedly different nucleotide sequences and the insertion of novel CDSs (i.e., *SacB*, which we predicted to encode a polysaccharide phosphotransferase, and rfbC/D which encodes a TDP-deoxymannose-dehydratase); (iii) and the *oatC* gene (that encodes polysialic acid-specific O-acetyltransferase), which was interrupted by the insertion of a mobile element of the IS4 transposase family. Search in the GenBank database demonstrated that the *SacB* gene was present only in the genome of the carriage *N. menigitidis* strain alpha275 (Genbank AM889138), but not in pathogenic strains. In addition, we found this gene also in *Neisseria mucosa*, suggesting that the rearranged region of the capsule locus in K1207 might have been acquired by horizontal gene transfer from nonpathogenic meningococci or other commensal *Neisseria* species.

The CDS content of the S0108 genome showed a high degree of similarity with FAM18 genome, with the exception of a large inversion in the capsule locus of a region of approximately 20 Kb included between the duplicated *rfbC* and *rfbC2* genes (Figures 2 and 3). In addition, the nucleotide sequence of the *rfbC2* gene in the FAM18 genome was different from the corresponding gene in K1207 and S0108 genomes.

## Discussion

In this study, an approach based on whole genome sequencing by NGS technology, comparative genomics, and gene function prediction was set up and applied to investigate *N. meningitidis* isolates, collected during an outbreak, that appeared identical by standard molecular typing methods based on PFGE, MLST, and *porA* VR1/VR2 sequencing. Whole genome sequencing demonstrated genetic changes between the fully sequenced isolates: then, a specific PCR based strategy applied to other samples from the same outbreak, followed by Sanger sequencing, allowed to distinguish outbreak cases from other temporal and geographically proximate but unrelated cases. This approach was designed in order to be feasible with a small budget. Nonetheless, the continuous decrease of sequencing costs, due to the improvement in the throughput of existing sequencing platforms and the simultaneous development of new technologies, will probably foster a wider employ of next generation sequencing in diagnostics and surveillance activities.

Comparative genomics analysis led to the identification and characterization of genetic differences among strains that might have been relevant for virulence and pathogenicity. In fact, in comparison with the *N. meningitidis* serogroup C ST-11/ET-37 reference strain FAM18 and with the S0108 isolate, the hyper-virulent K1207 outbreak isolate had marked changes in the capsule locus, consistent with recombination and horizontal acquisition of a set of genes involved in capsule biosynthesis from other meningococci. Although experimental verification is required, we hypothesize that these genetic changes might have enhanced transmissibility and invasiveness or even changed antigens involved in host immunity. In this regard, acquisition of novel genotypes at antigen-encoding loci has been reported to be probably the mechanism of emergence of hyper-virulent *N. meningitidis* ST-11 complex strains [22].

Homologous recombination and horizontal gene transfer are quite common in pathogenic *N. meningitidis* strains [23] and frequently affect virulence genes, including the capsule biosynthesis locus which may cause capsule switching [24], genes targeted by vaccines, and antibiotic susceptibility genes [25]. Considering this high variability, the conventional genotyping methods that are used to investigate outbreaks and to monitor the circulation of new variants of hyper-virulent strains may not be accurate enough. Several recent studies have demonstrated the power of whole genome sequencing by NGS technology to improve the identification of the geographical and evolutionary origin of an outbreak and to distinguish cases that belong to the outbreak from cases that do not [26].

The application of NGS technologies for the investigation of outbreaks in real time is becoming feasible thanks to the development of semi-automatic pipelines, as proposed in some recent publications [27-29]. In particular, Vogel *et al*. [27] demonstrated how to apply NGS in clinical practice, focusing on target genes of conventional typing methods (i.e., MLST, *porA* and *fetA* typing) and antimicrobial resistance gene testing (i.e., *penA* and *rpoB*). This approach can be expanded, as shown by Jolley *et al. *[28,29], on different subsets of genes, including the standard MLST loci, the ribosomal genes, personalized lists of specific loci, and whole sets of loci annotated on a complete reference genome. Such tools, which come along web platforms and interactive easy-to-use interfaces, are certainly essential instruments that will allow the introduction of whole genome sequencing in diagnostic routine. Instead, a limitation of their application in research is the capability to identify new determinants, not yet known and annotated [3]. The function prediction step integrated in our approach can provide valuable information to improve our understanding on unknown features, such as horizontal gene transfer and genome rearrangements. Though requiring experimental validation, these predictions could be useful to drive the experimental design and produce annotations: this new information will, on the one hand, enrich public databases and increase our knowledge about bacterial biodiversity and pathogenic mechanisms, and on the other hand it will be available for future routine diagnostic access.

## Conclusion

In conclusion, this study represents a practical example on the application of NGS technology to the investigation of an outbreak and how this technology may be helpful in the identification of outbreak cases with a direct clinical impact on the containment measures being taken. This study also shows how the avalanche of genomic information provided by NGS technology and bioinformatics analysis may contribute to our understanding of human pathogens.

## Competing interests

The authors declare that they have no competing interests.

## Authors’ contributions

EF carried out the wet lab procedures; EL, ST, FF, MF, and BDC performed the bioinformatics analyses; RM, GP and LB conceived the study and helped in its design and coordination; LB, ST and EL wrote the manuscript. All authors read and approved the final manuscript.

## Pre-publication history

The pre-publication history for this paper can be accessed here:

http://www.biomedcentral.com/1471-2334/13/554/prepub

## Supplementary Material

Additional file 1: Figure S1 GO function predictions for proteins specific to one of the two isolates. Complete list of GO terms predicted with Argot2 webserver [30]. Click here for file

## References

[B1] Barzon L Lavezzo E Costanzi G Franchin E Toppo S Palù G Next-generation sequencing technologies in diagnostic virology J Clin Virol 2013 13 00086 3 doi: S1386-6532 10.1016/j.jcv.2013.03.003 23523339

[B2] Barzon L Lavezzo E Militello V Toppo S Palù G Applications of next-generation sequencing technologies to diagnostic virology Int J Mol Sci 2011 12 7861 7884 10.3390/ijms12117861 22174638 PMC3233444

[B3] Didelot X Bowden R Wilson DJ Peto TE Crook DW Transforming clinical microbiology with bacterial genome sequencing Nat Rev Genet 2012 13 601 612 22868263 10.1038/nrg3226 PMC5049685

[B4] Kahvejian A Quackenbush J Thompson JF What would you do if you could sequence everything? Nat Biotechnol 2008 26 1125 1133 10.1038/nbt1494 18846086 PMC4153598

[B5] Kostic AD Ojesina AI Pedamallu CS Jung J Verhaak RG Getz G Meyerson M PathSeq: software to identify or discover microbes by deep sequencing of human tissue Nat Biotechnol 2011 29 393 396 10.1038/nbt.1868 21552235 PMC3523678

[B6] Frasson I Lavezzo E Franchin E Toppo S Barzon L Cavallaro A Richter SN Palù G Antimicrobial treatment and containment measures for an extremely drug-resistant Klebsiella pneumoniae ST101 isolate carrying pKPN101-IT, a novel fully sequenced bla(KPC-2) plasmid J Clin Microbiol 2012 50 3768 3772 10.1128/JCM.01892-12 22972824 PMC3486238

[B7] Ferro A Baldo V Cinquetti S Corziali P Gallo G Lustro G Paludetti P Menegon T Baldovin T Palù G Trivello R Outbreak of serogroup C meningococcal disease in Veneto region, Italy Euro Surveill 2008 13 2 8008 18445389

[B8] Fazio C Neri A Tonino S Carannante A Caporali MG Salmaso S Mastrantonio P Stefanelli P Characterisation of Neisseria meningitidis C strains causing two clusters in the north of Italy in 2007 and 2008 Euro Surveill 2009 14 16 19179 19389338

[B9] Maiden MC Bygraves JA Feil E Morelli G Russell JE Urwin R Zhang Q Zhou J Zurth K Caugant DA Feavers IM Achtman M Spratt BG Multilocus sequence typing: a portable approach to the identification of clones within populations of pathogenic microorganisms Proc Natl Acad Sci USA 1998 95 3140 3145 10.1073/pnas.95.6.3140 9501229 PMC19708

[B10] Suker J Feavers IM Achtman M Morelli G Wang JF Maiden MC The porA gene in serogroup A meningococci: evolutionary stability and mechanism of genetic variation Mol Microbiol 1994 12 253 265 10.1111/j.1365-2958.1994.tb01014.x 8057850

[B11] Lavezzo E Toppo S Barzon L Cobelli C Di Camillo B Finotello F Franchin E Peruzzo D Toffolo GM Trevisan M Palù G Draft genome sequences of two Neisseria meningitidis serogroup C clinical isolates J Bacteriol 2010 192 5270 5271 10.1128/JB.00789-10 20675487 PMC2944513

[B12] Finotello F Lavezzo E Fontana P Peruzzo D Albiero A Barzon L Falda M Di Camillo B Toppo S Comparative analysis of algorithms for whole-genome assembly of pyrosequencing data Brief Bioinform 2012 13 269 280 10.1093/bib/bbr063 22021898

[B13] Li W Jaroszewski L Godzik A Clustering of highly homologous sequences to reduce the size of large protein database Bioinformatics 2001 17 282 283 10.1093/bioinformatics/17.3.282 11294794

[B14] Li W Jaroszewski L Godzik A Tolerating some redundancy significantly speeds up clustering of large protein databases Bioinformatics 2002 18 77 82 10.1093/bioinformatics/18.1.77 11836214

[B15] Delcher AL Bratke KA Powers EC Salzberg SL Identifying bacterial genes and endosymbiont DNA with Glimmer Bioinformatics 2007 23 673 679 10.1093/bioinformatics/btm009 17237039 PMC2387122

[B16] Altschul SF Madden TL Schäffer AA Zhang J Zhang Z Miller W Lipman DJ Gapped BLAST and PSI-BLAST: a new generation of protein database search programs Nucleic Acids Res 1997 25 3389 3402 10.1093/nar/25.17.3389 9254694 PMC146917

[B17] Fontana P Cestaro A Velasco R Formentin E Toppo S Rapid annotation of anonymous sequences from genome projects using semantic similarities and a weighting scheme in gene ontology PLoS One 2009 4 e4619 10.1371/journal.pone.0004619 19247487 PMC2645684

[B18] Falda M Toppo S Pescarolo A Lavezzo E Di Camillo B Facchinetti A Cilia E Velasco R Fontana P Argot2: a large scale function prediction tool relying on semantic similarity of weighted Gene Ontology terms BMC Bioinformatics 2012 13 Suppl 4 S14 10.1186/1471-2105-13-S4-S14 22536960 PMC3314586

[B19] Radivojac P Clark WT Oron TR Schnoes AM Wittkop T Sokolov A Graim K Funk C Verspoor K Ben-Hur A Pandey G Yunes JM Talwalkar AS Repo S Souza ML Piovesan D Casadio R Wang Z Cheng J Fang H Gough J Koskinen P Törönen P Nokso-Koivisto J Holm L Cozzetto D Buchan DW Bryson K Jones DT Limaye B A large-scale evaluation of computational protein function prediction Nat Methods 2013 10 221 227 10.1038/nmeth.2340 23353650 PMC3584181

[B20] Elias J Vogel U IS1301 fingerprint analysis of Neisseria meningitidis strains belonging to the ET-15 clone J Clin Microbiol 2007 45 159 167 10.1128/JCM.01322-06 17093016 PMC1828961

[B21] Vogel U Claus H Frosch M Caugant DA Molecular basis for distinction of the ET-15 clone within the ET-37 complex of Neisseria meningitidis J Clin Microbiol 2000 38 941 10722324 10.1128/jcm.38.2.941-942.2000 PMC86260

[B22] Jolley KA Wilson DJ Kriz P McVean G Maiden MC The influence of mutation, recombination, population history, and selection on patterns of genetic diversity in Neisseria meningitidis Mol Biol Evol 2005 22 562 569 15537808 10.1093/molbev/msi041

[B23] Schoen C Tettelin H Parkhill J Frosch M Genome flexibility in Neisseria meningitidis Vaccine 2009 27 Suppl 2 B103 B111 19477564 10.1016/j.vaccine.2009.04.064 PMC3898611

[B24] Swartley JS Marfin AA Edupugnanti S Liu LJ Cieslak P Perkins B Wenger JD Stephens DS Capsule switching of Neisseria meningitidis Proc Natl Acad Sci USA 1997 94 271 276 10.1073/pnas.94.1.271 8990198 PMC19312

[B25] Hao W Ma JH Warren K Tsang RS Low DE Jamieson FB Alexander DC Extensive genomic variation within clonal complexes of Neisseria meningitidis Genome Biol Evol 2011 3 1406 1418 10.1093/gbe/evr119 22084315 PMC3242501

[B26] Didelot X Genomic analysis to improve the management of outbreaks of bacterial infections Expert Rev Anti Infect Ther 2013 11 335 337 10.1586/eri.13.15 23566141

[B27] Vogel U Szczepanowski R Claus H Jünemann S Prior K Harmsen D Ion Torrent Personal Genome Machine sequencing for genomic typing of Neisseria meningitidis for rapid determination of multiple layers of typing information J Clin Microbiol 2012 50 1889 1894 10.1128/JCM.00038-12 22461678 PMC3372157

[B28] Jolley KA Maiden MC Automated extraction of typing information for bacterial pathogens from whole genome sequence data: Neisseria meningitidis as an exemplar Euro Surveill 2013 18 20379 23369391 10.2807/ese.18.04.20379-en PMC3977036

[B29] Jolley KA Hill DM Bratcher HB Harrison OB Feavers IM Parkhill J Maiden MC Resolution of a meningococcal disease outbreak from whole-genome sequence data with rapid Web-based analysis methods J Clin Microbiol 2012 50 3046 3453 10.1128/JCM.01312-12 22785191 PMC3421817

[B30] A.r.g.o.t.2, Functional annotation of proteins using the semantic similarity in the Gene Ontology http://www.medcomp.medicina.unipd.it/Argot2

